# A retrospective propensity score-matched analysis of oncological and functional outcomes of submental island flap versus radial forearm free flap for oral cavity cancer reconstruction

**DOI:** 10.1186/s12903-024-03955-x

**Published:** 2024-02-05

**Authors:** Pichit Sittitrai, Donyarat Ruenmarkkaew, Chananchida Kumkun, Chonticha Srivanitchapoom

**Affiliations:** 1 Department of Otolaryngology, Faculty of Medicine, Chiang Mai University, Chiang Mai, Thailand; 2 Otolaryngology unit, Phayao Hospital, Phayao, Thailand

**Keywords:** Oral cancer, Reconstruction, Pedicled flap, Free flap

## Abstract

**Background:**

This retrospective study aims to compare the oncological and functional outcomes of the submental island flap versus the radial forearm free flap used for oral cavity cancer reconstruction after minimizing differences in baseline characteristics.

**Methods:**

Propensity scores for each oral cavity cancer patient who underwent surgical resection and immediate reconstruction with a submental island flap or a radial forearm free flap with a flap size ≤ 60 cm^2^ between October 2008 and December 2021 were generated based on the likelihood of being selected given their baseline characteristics. Patients were matched using a 1:1 nearest-neighbor approach.

**Results:**

The final matched-pair analysis included 51 patients in each group. The 5-year overall survival, disease-specific survival, and locoregional control rates were 70.1% and 64.8% (*p* = 0.612), 77.3% and 83.7% (*p* = 0.857), and 76.1% and 73.3% (*p* = 0.664), respectively, for the submental island flap group and the radial forearm free flap group. Speech and swallowing functions were comparable between groups. However, there were significantly lower rates of complication associated with both donor and recipient sites in the submental island flap group, and also the duration of hospital stays and hospital costs were significantly lower in these patients. A subgroup analysis of patients in which the reconstruction was carried out using the submental island flap procedure revealed that in selected cases, the presence of clinically and pathologically positive level I lymph nodes did not affect survival or locoregional control rates.

**Conclusions:**

Although this study was not randomized, the matched-pair analysis of surgically treated oral cavity cancer patients showed that submental island flap reconstruction is as effective as radial forearm free flap reconstruction with regard to oncological and functional outcomes with lower complication rates, hospital stay, and hospital costs. This flap can be safely and effectively performed in selected cases with a clinical level I lymph node smaller than 1.5 cm and no signs of extranodal extension.

## Background

Surgical resection is the mainstay of treatment of oral cavity cancer, followed by adjuvant radiotherapy and chemotherapy when indicated [1]. However, soft tissue reconstruction of an oral defect after tumor extirpation is a major challenge, the aims being to provide adequate wound healing and successful functional rehabilitation. In addition, the concepts related to reconstruction should be balanced between maximal efficacy and outcome as well as minimal complication and cost [2].

Due to their reliability and adaptability, free flaps have been referred to as the gold standard for reconstructing defects after surgical resection in the head and neck, including oral cavity cancer [3]. However, not all defects require a free flap to achieve good outcomes, and not every case is suitable for a microvascular procedure. Therefore, regional pedicled flaps may be helpful in treating patients with coexisting morbidities, a short life expectancy, poor vascularity of the recipient site, and in other specific circumstances, including lack of microsurgical facilities and financial issues [4–7].

The radial forearm free flap is a popular choice for reconstruction after resection of oral cavity cancer because of its versatility and good functional outcomes in combination with low flap loss and complication rates [8, 9]. In comparison, the submental island flap is an effective functional local flap for oral cavity reconstruction, given its reliability, low donor site morbidity, and short operative time [10].

Studies have been carried out comparing treatment outcomes between submental island flap and radial forearm free flap reconstruction in the head and neck [11], oral tongue [12, 13], and oral cavity defects [4, 14, 15]. The matched design approach was conducted in 2 studies, however, one was matched with a similar ablative volume defect of 12 patients in each group and included both radial and ulnar forearm free flaps [14]. The second was matched with the same intraoral region and analogous T-status, which included only patients with clinical and pathological N0 (specifically level I cervical lymph node) and a defect size smaller than 6 × 4 cm [15]. However, a submental island flap can be used safely in selected patients with level I lymph node metastases when the flap is meticulously harvested, with a skin paddle potentially as large as 18 × 7 cm [8, 12]. Also, both studies did not define the matching method.

This study aims to compare oncological and functional outcomes in oral cavity squamous cell carcinoma patients who underwent surgical resection followed by soft tissue reconstruction with a submental island flap or a radial forearm free flap. To create a statistically balanced pair of patients from a retrospective data set, we performed the propensity score-matched analysis by using potential confounding baseline characteristics as matching criteria to reduce the bias of flap selection.

## Materials and methods

### Patient selection

We conducted a retrospective study of patients aged more than 18 years with squamous cell carcinoma of the oral cavity who underwent surgical resection and immediate reconstruction with a submental island flap or a radial forearm free flap between October 2008 and December 2021, with follow-up recorded up to December 2022. The medical records were accessed from the archives of our Department. Patients with recurrent oral cavity cancer or distant metastasis, those who required a segmental mandibulectomy, or patients who had undergone previous neck surgery or radiotherapy, or had a history of cancer elsewhere in the body, were excluded from the study. The patient data, with confidentiality and anonymity, was stored in an encrypted file on a personal computer and could only be accessed and used by the researchers for authorized purposes. The data file will be permanently deleted within 1 year after the manuscript has been published.

**Fig. 1:**
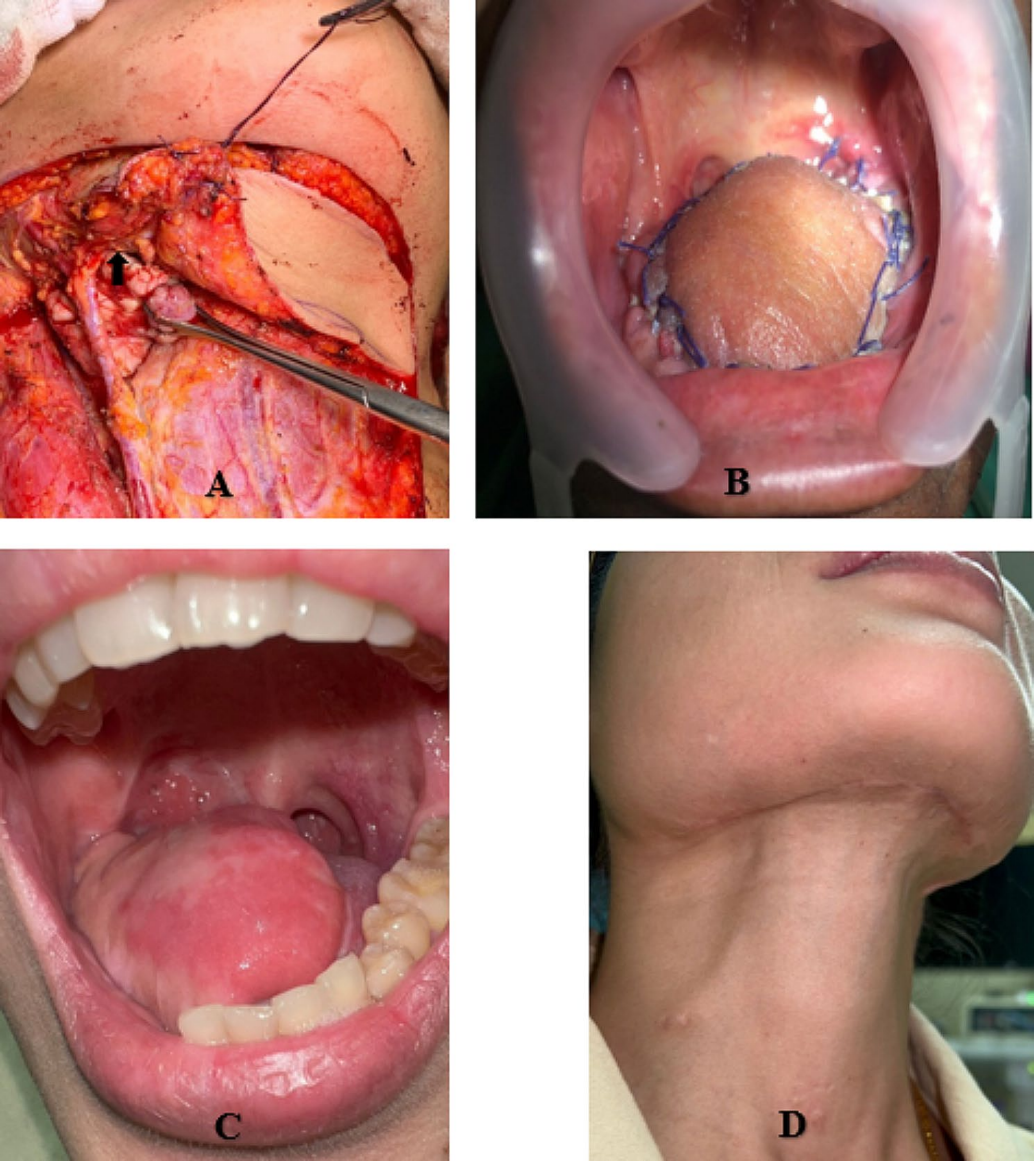
Submental island flap reconstruction following oral tongue cancer resection; harvested flap with submental vessels (black arrow) (**A**), and the flap was transferred to the oral cavity (**B**), intraoral flap 12 months after surgery (**C**), and scar at neck (**D**)

**Fig. 2:**
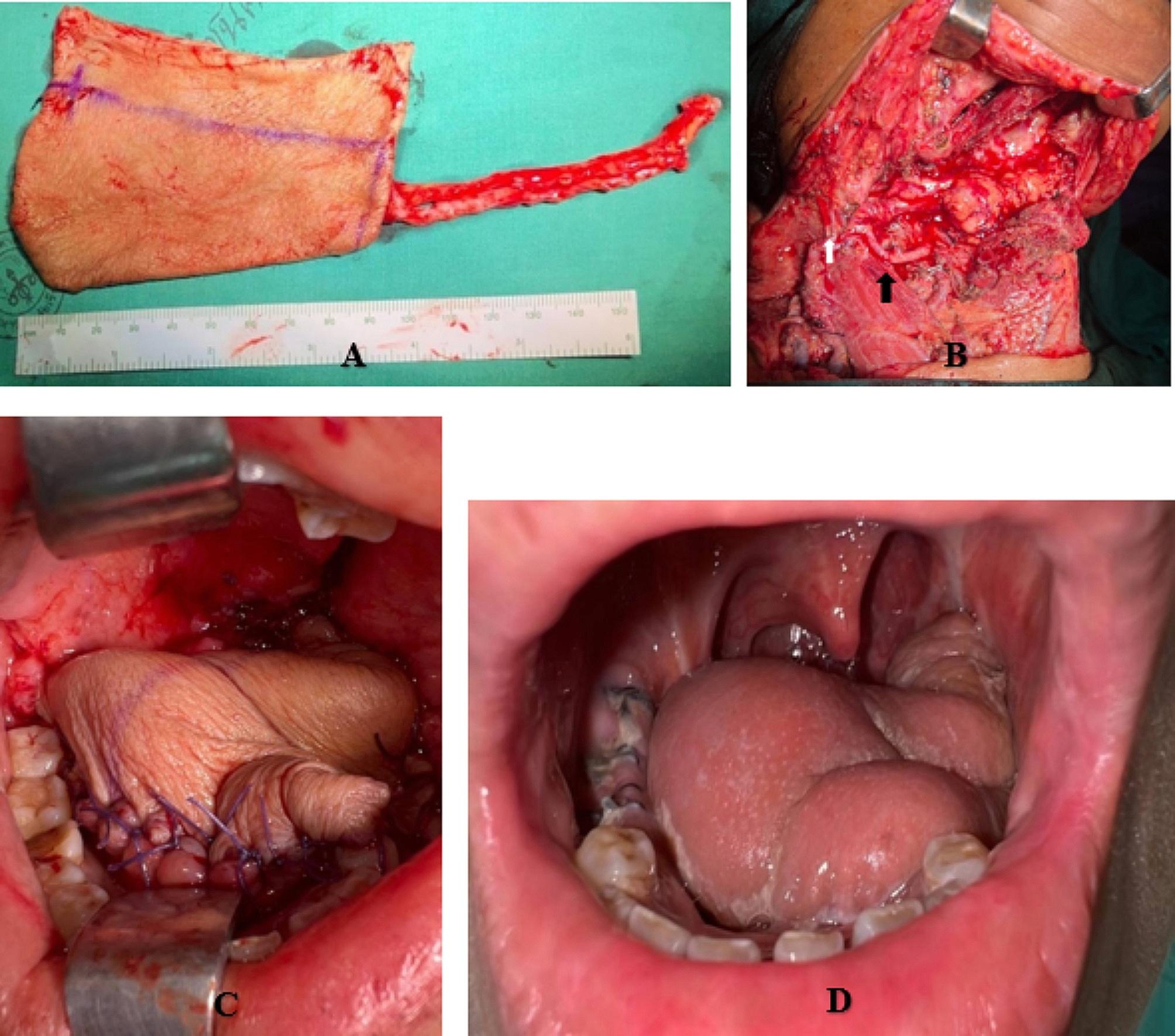
Radial forearm free flap reconstruction following oral tongue cancer resection; harvested flap (**A**), arterial anastomosis between the superior thyroid artery and the radial artery (black arrow), and venous anastomosis between the external jugular vein and the cephalic vein (white arrow) (**B**), the flap was transferred to the oral cavity (**C**), and intraoral flap 12 months after surgery (**D**)

All patients underwent selective or comprehensive neck dissection and either unilateral or bilateral neck dissection, depending on the clinical status of the cervical lymph node. Patients with a primary tumor extending through the mylohyoid muscle or with a level I cervical lymph node in the submental flap area with a sign of extranodal extension of cancer or node larger than 1.5 cm in diameter detected preoperatively were contraindicated for a reconstruction using a submental island flap but not for a radial forearm free flap [16]. During the operation, the level I lymph node was meticulously dissected and sent for permanent pathological examination. A suspicious lymph node with the characters mentioned above detected within the area of the submental island flap was sent for a frozen section. If the result of the frozen section was a metastatic lymph node, a radial forearm free flap reconstruction and a comprehensive neck dissection would be performed. However, if there were positive lymph nodes along the ipsilateral vascular pedicle, the contralateral vessels would be the vascular supply of the submental island flap. Otherwise, the decision of treatment choice and method of reconstruction was made following a multidisciplinary discussion that considered the patient’s preference, comorbidities, and performance status. All procedures were performed by the two senior authors (Figs. 1 and 2). Postoperative adjuvant therapies, including radiotherapy or chemoradiotherapy, were considered if the tumors were of an advanced stage (stages III-IV), were close to or had invaded surgical margins, or exhibited an extracapsular extension of the lymph nodes.

After completion of treatment, follow-up protocols, in line with the NCCN Clinical Practice Guidelines in Oncology [17], follow-up included clinical evaluation every 1–3 months in the first year, every 2–6 months in the second year, every 4–8 months in the third to fifth years, and every 12 months after the fifth year. CT scan or MRI was performed at 8–12 weeks or PET/CT at 12–16 weeks after treatment, and then a CT scan was performed annually.

### Propensity matched-pair analysis

The data of 146 patients were obtained: 52 reconstructions with a submental island flap and 94 reconstructions with a radial forearm free flap. As the largest skin paddle size of the submental island flap was 60 cm^2^, and that of the radial forearm free flap was 84 cm^2^, to decrease bias selection from the size, 13 patients with a flap size larger than 60 cm^2^ were excluded before performing the analysis.

**Fig. 3:**
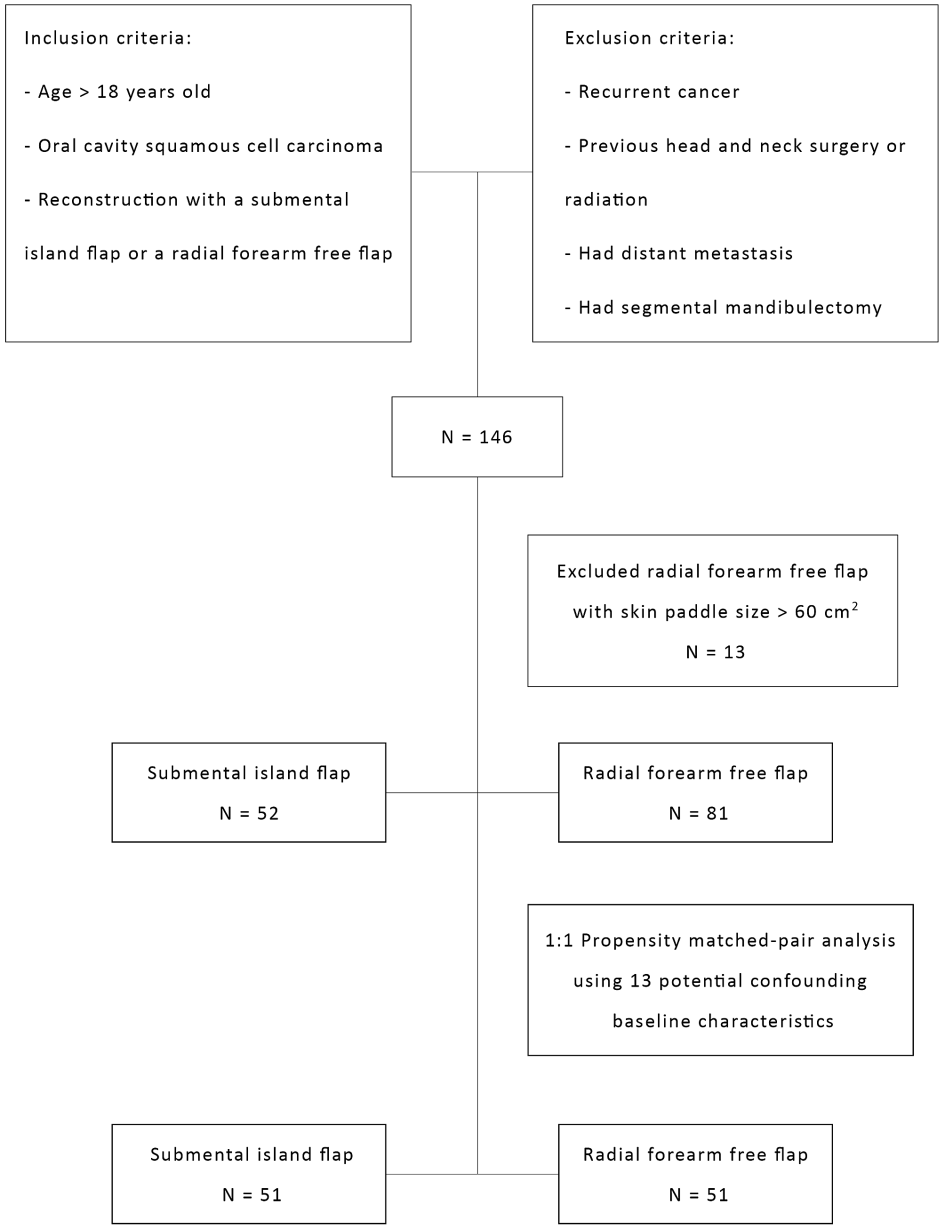
Flow diagram of enrollment and matched pair of the study group

The remaining 133 patients were included for a propensity-score matched-pair analysis to minimize the bias related to flap selection and assignment. The propensity score matching approach involved two steps. In the first step, the likelihood that a patient would receive a submental island flap was assessed using a logistic regression model as a function of age, gender, history of smoking and alcohol usage, underlying diseases (diabetes mellitus and chronic obstructive pulmonary disease), Eastern Cooperative Oncology Group Performance Status (ECOG PS), subsites of the oral cavity, primary tumor stage, nodal stage, clinical status of level I lymph node, type of neck dissection, and flap size. From this regression, the predicted probability of receiving a submental island flap, or propensity score, was calculated for each patient. In the second step, patients were matched 1:1 from both groups based on the propensity scores with a caliper width of 0.25 SD. In the case of more than two matches, one pair was picked randomly from among all potential matches. Cases without a matched control were excluded (Fig. 3).

### Outcomes

The primary outcomes of the study were the oncological results of the whole cohort, including overall survival (OS), disease-specific survival (DSS), and locoregional control (LRC). These outcomes would be compared to historical data. In addition, the oncological safety of using the submental island flap reconstruction in oral cavity cancer has been a cause for concern from the possibility of metastatic lymph nodes in level I. However, with strict indications and a meticulously surgical procedure, we expected no differences in the outcomes from subgroup analysis between the submental island flap and the radial forearm free flap. OS was defined as the time from surgery until the date of death from any cause. DSS was defined as the time from surgery until the date of death from oral cavity cancer. In case of no death from oral cavity cancer, the observation was censored at the last follow-up visit or the date of death from other causes. LRC was defined as the time from surgery until the absence of any progression of the primary tumor or occurrence of cervical lymph node metastases. Patients were censored at the moment of their last visit, when they died from causes other than oral cavity cancer, or when they were lost to follow-up.

Secondary outcomes were speech and swallowing functions, which were assessed using a scoring system, as shown in Table 1 [4, 12]. The plan was to include evaluation of every patient for at least 1 year after treatment. In addition, surgical complications, duration of hospital stay, and hospital costs were reviewed.

**Table 1:** Postoperative functional results of speech and swallowing scores

Score	Speech	Swallowing
**5**	all speech is understood (excellent)	full diet
**4**	speech is sometimes not understood (good)	soft diet
**3**	speech can be understood when conversational content is already known (fair)	liquid diet
**2**	speech can sometimes be understood (poor)	combined oral and feeding tube
**1**	nothing is understood (bad)	exclusively by feeding tube

All procedures contributing to this work complied with the ethical standards of the relevant national and institutional guidelines on human experimentation and with the Declaration of Helsinki 1975, revised in 2013. The study was approved by the Institutional Research Ethics Committees, approved on 25 October 2021 (approval number: 453/2021) and 5 January 2023 for additional data collection (approval number: 024/2023). The committees waived the need for participant consent for this retrospective study.

### Statistical analysis

Categorical variables were summarized as frequencies (%). Continuous variables were checked for normality and summarized as mean ± standard deviation if normally distributed or as median (interquartile range) if not. Pearson’s chi-square test or Fisher exact test were used to compare categorical variables with frequencies larger than 5 or smaller than 5, respectively. A Student’s t-test was used to compare continuous variables which were normally distributed, and the Mann-Whitney U test was used for comparing continuous variables with a non-normal distribution. The Kaplan-Meier curve was used to analyze the survival data, and differences between groups were conducted using the log-rank test. Multivariate Cox proportional hazards model analyses were carried out to study the influence of clinical and pathological parameters on survival outcomes. Propensity score-matched analysis by means of logistic regression and 1:1 matching was performed based on the propensity score of each patient. The quality of the match was assessed by recalculating the standardized mean difference of each variable in the matched sample until a balance was achieved. For all statistical analyses, a p-value less than 0.05 was considered statistically significant. The analyses were performed using the SPSS software package version 22.0 (SPSS Inc., Chicago, IL, USA).

## Results

### Patient characteristics

The final matched pair analysis included 102 patients (51 patients in the submental island flap group and 51 patients in the radial forearm free flap group) with a median follow-up time of 61 months (range, 5-147 months). Patient characteristics before and after matching are listed in Table 2. There were significant differences in the N stage, the number of patients with pathologically positive level I lymph nodes, type of neck dissection, and operative time between the two groups before matching. However, only operative time was significantly different after matching (197 min in the submental island flap group and 450 min in the radial forearm free flap group, *p* < 0.001).

**Table 2:** Patient baseline characteristics before and after matching

Variables	Before matching *n* = 133			After matching *n* = 102		
	SMF^a^ *n* = 52 (%)	RFF^b^ *n* = 81 (%)	p-value	SMF^a^ *n* = 51 (%)	RFF^b^ *n* = 51 (%)	p-value
**Age** (mean ± SD (range); years)	59.6 ± 11.1 (33–84)	56.9 ± 10.9 (32–80)	0.186	59.2 ± 10.9 (33–84)	58.6 ± 10.7 (32–80)	0.791
**Sex**						
- Male - Female	32 (61.5) 20 (38.5)	52 (64.2) 29 (35.8)	0.854	32 (62.7) 19 (37.3)	30 (58.8) 21 (41.2)	0.839
**Smoke**						
- Yes - No	34 (65.4) 18 (34.6)	52 (64.2) 29 (35.8)	1.000	33 (64.7) 18 (35.3)	34 (66.7) 17 (33.3)	1.000
**Alcohol**						
- Yes - No	33 (63.5) 19 (36.5)	53 (65.4) 28 (34.6)	0.854	34 (65.4) 18 (34.6)	34 (65.4) 18 (34.6)	1.000
**DM**						
- Yes - No	7 (13.5) 45 (86.5)	9 (11.1) 72 (88.9)	0.786	7 (13.7) 44 (86.3)	7 (13.7) 44 (86.3)	1.000
**COPD**						
- Yes - No	14 (26.9) 38 (73.1)	19 (23.5) 62 (76.5)	0.684	14 (27.5) 37 (72.5)	11 (21.6) 40 (78.4)	0.646
**ECOG PS** ^b^						
− 0 or 1 − 2	41 (78.8) 11 (21.2)	69 (85.2) 12 (14.8)	0.358	40 (78.4) 11 (21.6)	42 (82.4) 9 (17.6)	0.804
**Primary site location**						
- Tongue - Floor of the mouth - Buccal mucosa - Alveolar ridge - Retromolar trigone	29 (55.8) 14 (26.9) 5 (9.6) 3 (5.8) 1 (1.9)	48 (59.3) 12 (14.8) 12 (14.8) 6 (7.4) 3 (3.7)	0.513	29 (56.9) 14 (27.5) 4 (7.8) 3 (5.9) 1 (2.0)	27 (52.9) 9 (17.6) 10 (19.6) 4 (7.8) 1 (2.0)	0.394
**T stage**						
**-** T1 - T2 - T3 - T4	1 (1.9) 13 (25) 20 (38.5) 18 (34.6)	- 15 (18.5) 31 (38.3) 35 (42.3)	0.422	1 (2.0) 13 (25.5) 20 (39.2) 17 (33.3)	- 9 (17.6) 21 (41.2) 21 (41.2)	0.582
**N stage**						
- N0 - N1 - N2 - N3	22 (42.3) 12 (23.1) 16 (30.8) 2 (3.8)	18 (22.2) 12 (14.8) 41 (50.6) 10 (12.3)	**0.011** ^f^	21 (41.2) 12 (23.5) 16 (31.4) 2 (3.9)	15 (29.4) 8 (15.7) 22 (43.1) 6 (11.8)	0.206
**Clinically + Level I lymph node**	18 (34.6)	41 (50.6)	0.077	18 (35.3)	21 (41.2)	0.684
**Pathologically + Level I lymph node**	13 (25)	34 (41.9)	0.048^f^	15 (29.4)	20 (39.2)	0.404
**Pathological ENE**	4 (7.7)	11 (13.6)	0.295	3 (5.9)	6 (11.8)	0.487
**Neck dissection** **Side**						
- Unilateral - Bilateral	18 (34.6) 34 (65.4)	23 (28.4) 58 (71.6)	0.564	17 (33.3) 34 (66.7)	19 (37.3) 32 (62.7)	0.836
**Type**						
- Selective - Comprehensive	22 (42.3) 30 (57.7)	18 (22.4) 63 (77.8)	0.020^f^	21 (41.2) 30 (58.8)	15 (29.4) 36 (70.6)	0.300
**Flap size** (median (IQR);cm^2^)^**c**^	30 (24-35.8)	30 (28–42)	0.177	30 (24–36)	32 (28–42)	0.193
**Operation time** (mean ± SD (range);min)	196 ± 52 (80–340)	454 ± 84 (290–690)	< 0.001^f^	197 ± 53 (80–340)	450 ± 78 (290–595)	< 0.001^f^
**Pathological margin**						
- Clear - Close or Positive	20 (38.5) 32 (61.5)	27 (33.3) 54 (66.7)	0.581	20 (39.2) 31 (60.8)	18 (35.3) 33 (64.7)	0.838
**Postoperative treatment**						
- No - RT or CRT^e^	9 (17.3) 43 (82.7)	10 (12.3) 71 (87.7)	0.454	9 (17.6) 42 (82.4)	7 (13.7) 44 (86.3)	0.786

^a^ Submental island flap; ^b^ Radial forearm free flap; ^c^ The Kolmogorov-Smirnov normality test revealed that the data was not normally distributed (p-value < 0.05).; ^d^ Eastern Cooperative Oncology Group Performance Status; ^e^ Radiotherapy or Chemoradiotherapy; ^f^ Statistically significant

### Oncological outcomes

**Fig. 4:**
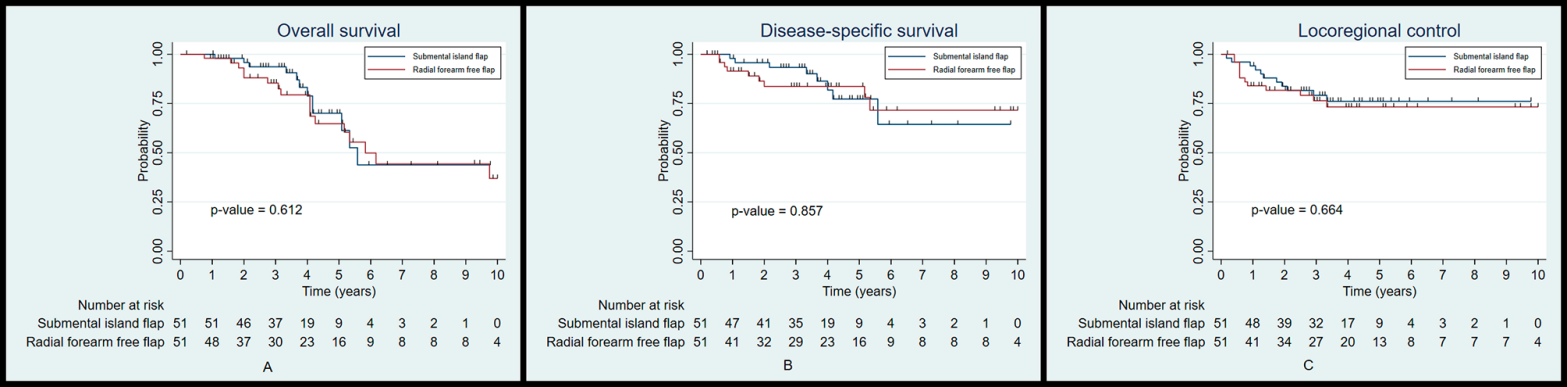
Kaplan-Meier plot for overall survival (**A**), disease-specific survival (**B**), and locoregional control (**C**) according to the type of flap reconstruction

The 5-year OS, DSS, and LRC rates between the patients reconstructed with a submental island flap and those reconstructed with a radial forearm free flap were 70.1% and 64.8% (*p* = 0.612; Fig. 4A), 77.3% and 83.7% (*p* = 0.857; Fig. 4B), and 76.1% and 73.3% (*p* = 0.664; Fig. 4C), respectively. The results showed that there were no significant differences found in the survival and locoregional control rates between the two groups.

The multivariate Cox proportional hazard regression model analysis was performed based on the type of flap reconstruction and clinicopathological variables for the oncological outcomes. The analysis revealed that the type of flap reconstruction did not affect the outcomes. However, ECOG PS 2 was a significant independent predictor of overall mortality (HR 6.74, 95% CI 1.89–23.95, *p* = 0.003). Furthermore, nodal stage N3 compared to N0 and pathologically positive level I lymph nodes were associated with an increased risk of locoregional recurrence (HR 1.85, 95% CI 1.14–5.14, *p* = 0.048, and HR 3.52, 95% CI 1.08–11.52, *p* = 0.038, respectively). However, no variable affecting disease-specific mortality was detected (Table 3). Notably, the type of flap reconstruction did not statistically influence the oncological outcomes. Concerning disease recurrence, no significant differences in local, regional, and distant organ recurrences were observed between the submental island flap group and the radial forearms free flap group (11.8% and 15.7%, *p* = 0.775, 13.7% and 9.8%, *p* = 0.769, and 7.8% and 15.7%, *p* = 0.357, respectively).

**Table 3:** Multivariate Cox proportional hazard regression model analysis based on reconstruction type and clinicopathological variables potentially affecting oncological outcomes

Variables	HR (95% CI)	p-value
**Overall mortality**		
ECOG PS^a^ 2	6.74 (1.89–23.95)	0.003^b^
**Locoregional recurrence**		
N0	Reference	
N1 N2 N3	1.38 (0.08–1.69) 1.25 (0.06–0.99) 1.85 (1.14–5.14)	0.203 0.826 0.048^b^
Pathologically positive level I lymph nodes	3.52 (1.08–11.52)	0.038^b^

^a^ Eastern Cooperative Oncology Group Performance Status; ^b^ Statistically significant

### Subgroup analysis

**Fig. 5:**
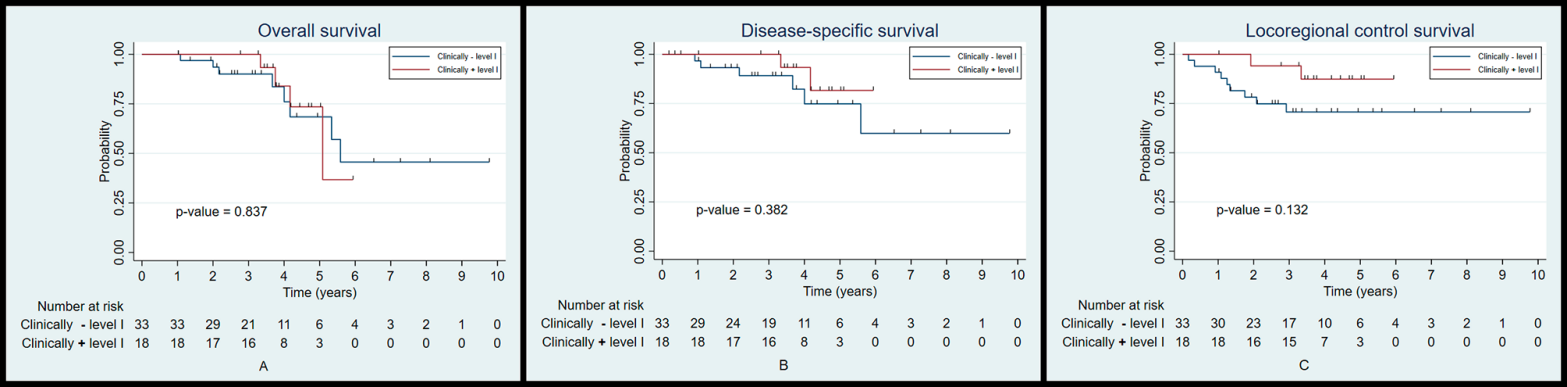
Kaplan-Meier plot for overall survival (**A**), disease-specific survival (**B**), and locoregional control (**C**) according to the clinical status of level I lymph nodes of the submental island flap subgroup

**Fig. 6:**
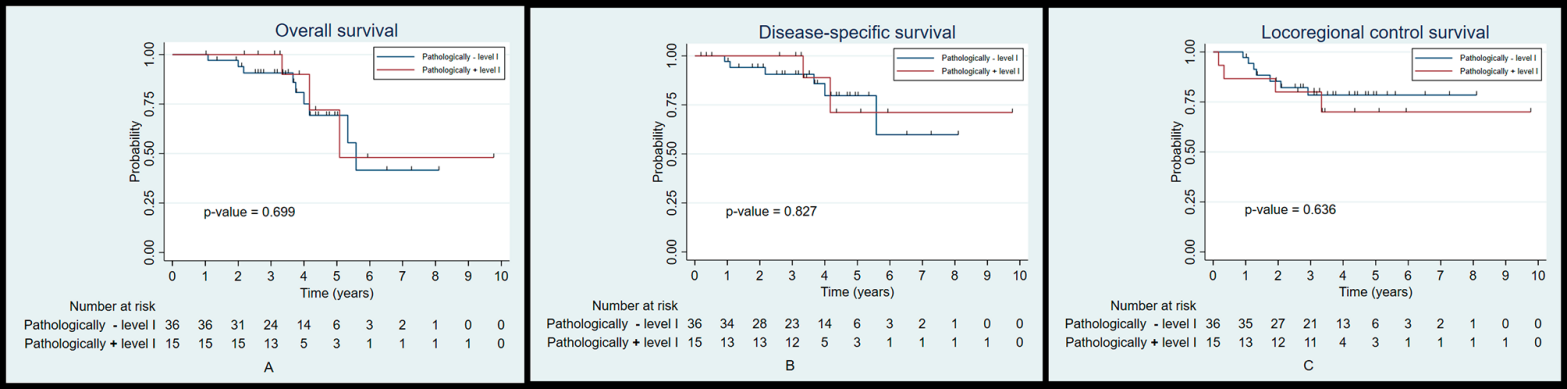
Kaplan-Meier plot for overall survival (**A**), disease-specific survival (**B**), and locoregional control (**C**) according to the pathological status of level I lymph nodes of the submental island flap subgroup

We performed a subgroup analysis of patients reconstructed with a submental island flap in association with level I cervical lymph node status on the oncological outcomes. The results demonstrated that there were no statistical differences in the 5-year OS, DSS, and LRC rates in patients with clinically negative or positive level I lymph nodes (68.4% and 73.5%, *p* = 0.837, 74.8% and 81.7%, *p* = 0.382, and 70.7% and 87.4%, *p* = 0.132, respectively) (Fig. 5A-C). Furthermore, pathologically negative or positive level I lymph nodes also did not affect the outcomes (69.3% and 72%, *p* = 0.699, 79.7% and 71.1%, *p* = 0.827, and 78.4% and 70%, *p* = 0.636, respectively) (Fig. 6A-C).

### Functional outcomes

There were no statistically significant differences in speech and swallowing functions between the two groups (Table 4). No patients had bad speech function, and most patients in both groups had excellent to good speech results (88.2% in the submental island flap group and 96.1% in the radial forearm free flap group, *p* = 0.269). In addition, most of the patients in both groups were able to take at least a soft diet (78.4% in the submental island flap group and 90.2% in the radial forearm free flap group, *p* = 0.172). However, obligated tube feeding was necessary in 1 patient (2%) in the submental island flap group.

**Table 4:** Speech and swallowing outcomes of patients

Functional outcomes	SMF^a^ *n* = 51 (%)	RFF^b^ *n* = 51 (%)	p-value
**Speech**			
- Excellent or Good - Fair or Poor	45 (88.2) 6 (11.8)	49 (96.1) 3 (3.9)	0.269
**Swallowing**			
- Full or Soft diet - Liquid diet or Feeding tube	40 (78.4) 11 (21.6)	46 (90.2) 5 (9.8)	0.172

^a^ Submental island flap; ^b^ Radial forearm free flap

### Surgical complications

Donor site complications were detected more frequently in the radial forearm free flap group (15.7% and 2%, *p* = 0.031). Bleeding at the wound edge was recorded in 1 patient in the submental island flap group on the first postoperative day. Suturing at the bleeding site under local anesthesia was successfully performed. There were no other records of donor site complications in the submental island flap group, including no marginal mandibular branch paralysis. The donor site defects in all cases were primarily closed, with pleasing cosmesis and without restricted neck extension. However, in the radial forearm free flap group, there was partial loss of the skin graft in 2 patients (3.9%), and arm function, including grip strength, pinch strength, and wrist movements, was restricted in 5 patients (9.8%). In addition, 1 patient with bleeding at the raw surface under the skin graft was detected on the first postoperative day and treated with cauterization (Table 5).

**Table 5:** Surgical complications, duration of hospital stay, and hospital costs of patients

	SMF^a^ *n* = 51 (%)	RFF^b^ *n* = 51 (%)	p-value
**Donor site complications**			
- No - Yes	50 (98.0)	43 (84.3)	0.031^d^
- Bleeding - Wound dehiscence - Partial loss of skin graft - Restricted neck/arm function	1 (2.0) - - -	1 (1.9) - 2 (3.9) 5 (9.8)	
**Recipient site complications**			
- No - Yes	43 (86.0)	34 (66.7)	0.034^d^
- Orocutaneous fistula - Wound dehiscence - Minor infection - Hematoma	- 4 (7.8) 2 (3.9) 2 (3.9)	3 (5.9) 7 (13.7) 7 (13.7) 2 (3.9)	
**Flap Complications**			
- No - Yes	46 (90.2)	44 (86.3)	0.760
- Partial flap loss - Revision of the anastomosis	5 (9.6) -	5 (9.6) 2 (3.9)	
**Hospital stay** (median (IQR);days)^**c**^	19 (15–24)	22 (17–30)	0.018^d^
**Hospital costs** (median (IQR);USD)^c^	4,098 (3,206-5,104)	6,243 (5,389-6,966)	< 0.001^d^

^a^ Submental island flap; ^b^ Radial forearm free flap; ^c^ The Kolmogorov-Smirnov normality test revealed that the data was not normally distributed (p-value < 0.05).; ^d^ Statistically significant

Recipient site complications were also detected significantly more frequently in the radial forearm free flap group (33.3% and 14%, *p* = 0.034). Orocutaneous fistula was recorded in 3 patients (5.9%) in the radial forearm free flap group, no such complication was recorded in the submental island flap group. The fistulas were treated with necrotic tissue debridement followed by re-suturing of the wounds. Other complications, including wound dehiscence, minor local infection, and small hematoma, were detected in both groups and treated successfully with wound re-suturing, local wound care, and wound reopening with collection removal (Table 5).

With regard to flap complications, neither group had total flap loss, but partial flap loss (partial epithelial loss) occurred in 5 patients in the submental island flap group. However, re-epithelialization was complete within four weeks with conservative management. Five patients in the radial forearm free flap group had less than 10% loss of the flap volume, and these patients were treated with tissue debridement and wound re-suturing. Radial forearm free flap congestion was detected in 2 patients, and the venous anastomosis was revised within 6 h of the first postoperative day (Table 5).

### Duration of hospital stay and hospital costs

The median duration of hospital stay was significantly shorter in the submental island flap group than in the radial forearm free flap group (19 days and 22 days, respectively; *p* = 0.018). Concerning median hospital costs during admission, submental island flap reconstruction was significantly less costly than those involving radial forearm free flap reconstruction (4,098 US Dollars and 6,243 US Dollars, respectively, *p* < 0.001) (Table 5).

## Discussion

The main goals of soft tissue reconstruction of the oral cavity following tumor resection are appropriate wound coverage and watertight closure to prevent surgical site infection and also facilitation of early oral intake, which may allow the patients to be discharged earlier and receive timely adjuvant therapy. Furthermore, adequate functional restoration improves the quality of life [2].

Microvascular free flaps have been the preferred option for soft tissue reconstruction of the oral cavity. However, free flap reconstruction requires substantial perioperative resources, which may not be available in many head and neck cancer centers [8]. In addition, the complex reconstruction with prolonged operative time and hospital stay may not be suitable in a situation such as during the recent COVID-19 pandemic. Therefore, a variety of pedicled flaps have been performed more frequently in the recent past [18].

This study is a single-institution cohort study comparing submental island flap and radial forearm free flap reconstruction in oral cavity cancer patients. Selection bias was evident in the initial unmatched dataset. This necessitated matching for potential confounders using propensity scoring. After 1:1 matching with 13 clinicopathological variables, we did not find any statistically significant differences in terms of 5-year OS, DFS, and LRC rates, as well as distant metastatic rate with regard to the flap reconstruction group in the 51 matched pairs. The 5-year OS, DFS, and LRC rates of both groups in this study (70.1% and 64.8%, 77.3% and 83.7%, and 76.1% and 73.3%, respectively) are in line with previous reports (56–83%, 74–87%, and 65–83%, respectively) [19–23].

The multivariate Cox proportional hazard regression model analysis showed that ECOG PS 2 (compared with ECOG PS 0–1) independently affected overall mortality. In addition, nodal stage N3 (compared with N0) and pathologically positive level I lymph nodes were associated with an increased risk of locoregional recurrence. Notably, in this regression analysis, the type of flap reconstruction did not affect mortality and locoregional recurrence.

Primary lymphatic drainage associated with oral cavity cancer usually involves the level I (submental and submandibular) lymph node group. Therefore, the oncological safety of using the submental island flap has been a cause for concern [12, 16]. However, the subgroup analysis of patients reconstructed with the submental island flap revealed that the clinical and pathological status of level I lymph node did not affect the 5-year OS, DSS, and LRC rates. Therefore, as previously mentioned, in patients with a clinical level I cervical lymph node smaller than 1.5 cm in diameter, with no clinical signs of extracapsular spread, who have level I lymph nodes carefully dissected during flap harvesting, submental island flap reconstruction can be effectively carried out without compromising survival and locoregional recurrence.

Most of the patients in both groups had excellent to good speech results (88.2% in the submental island flap group and 96.1% in the radial forearm free flap group). Furthermore, the majority of the patients were able to take at least a soft diet (78.4% in the submental island flap group and 90.2% in the radial forearm free flap group). Although 1 patient (2%) in the submental island flap group required prolonged feeding by tube, this rate is in line with previous reports (2-8.3%) [4, 14, 15]. The functional outcomes of oral cavity reconstruction are determined by the mobility and the volume of the reconstructed tongue. Submental island flaps provide good bulkiness, while radial forearm free flaps are pliable and can be designed to increase the flap volume, as can be seen in the beavertail modification. Therefore, speech and swallowing functions were slightly superior in the radial forearm free flap group. However, no statistical differences were observed between the groups.

Donor site complication rate was significantly higher in the radial forearm free flap group, including skin graft loss (3.9%) and restricted wrist function (9.8%), which were the major drawbacks associated with this flap. Recipient site complication rate was also detected more frequently in association with radial forearm free flap reconstruction, in particular the incidence of orocutaneous fistula, which was detected in 5.9% of cases (5–15% in previous reports) [4, 11, 12]. Notably, none of the patients who underwent reconstruction with a submental island flap developed this complication (0–5% in previous reports) [4, 11, 12]. This is potentially because the musculofascial component of the flap occludes the dead space and reinforces the watertight closure of the defect [12]. In addition, our results demonstrated that reconstruction using a submental island flap was significantly associated with shorter operative time and duration of hospital stay and lower hospital costs, all of which are distinctive advantages of this flap. Accordingly, the cost minimization may be beneficial in low- and middle-income countries.

From previous studies and a recent meta-analysis, survival and locoregional control rates between the submental island flap and the radial forearm free flap were unable to be directly compared. This was due to the short follow-up period and higher nodal burden in the free flap group because most studies either excluded clinically positive level I lymph nodes or did not report the status of this lymph node group [4, 8, 11–15]. However, with appropriate matching and an adequate follow-up period, our results demonstrated that the 5-year oncological outcomes of these two flaps are similar. In addition, in the submental island flap subgroup, clinically and pathologically positive level I cervical lymph nodes had no effect on the survival and locoregional control rate.

This study has some strengths compared to previous studies. The propensity score matching is an effective tool to control for confounding bias in a retrospective observational study and, when applied properly, can simulate random assignment subjects seen in a randomized trial. To our knowledge, this is the first study comparing the outcomes between the submental island flap and the radial forearm free flap using the propensity match pair analysis with 13 clinicopathological variable adjustments.

Some limitations should be considered when interpreting the results of the current study. First, this is a retrospective single-center study, which limits the collection of relevant data for this investigation. Second, despite the design of this study involving the propensity matched-pair analysis by several substantial matching criteria, individual differences in patients, surgeon’s preference, and surgical complication concerns still contribute to some uncertainty. Third, although one-to-many propensity score matching in a cohort study can yield higher precision than 1:1 matching at the cost of a slight increase in bias, it is not suitable for the limited sample size with the different ratio between groups less than 1:2 as ours [24]. And lastly, subjective speech and swallowing outcomes assessment may not be as accurate as both objective and subjective evaluations. Thus, a randomized, multicenter trial with comprehensive assessments should be studied in the future.

## Conclusion

Matched-pair analysis by propensity score matching of submental island flap and radial forearm free flap with a flap size ≤ 60 cm^2^ for the reconstruction of an oral cavity defect after cancer resection revealed no difference in terms of oncological and functional outcomes. However, the submental island flap reconstruction resulted in fewer donor and recipient sites morbidities, shorter operative time and duration of hospital stay, and lower hospital costs. In addition, this flap was shown to be safely performed in selected patients with a clinical level I cervical lymph node smaller than 1.5 cm in diameter, with no clinical signs of extracapsular spread.

## Data Availability

The datasets used and/or analyzed during the current study are available from the corresponding author on reasonable request.

## Abbreviations

CRT: Chemoradiotherapy
DSS: Disease-specific survival
ECOG: Eastern Cooperative Oncology Group Performance Status
IQR: Interquartile range
LRC: Locoregional control
OS: Overall survival
RFF: Radial forearm free flap
RT: Radiotherapy
SMF: Submental island flap
